# 217. Preliminary Results from a Clinical Trial Comparing the Efficacy of Cefixime Versus Penicillin G for the Treatment of Early Syphilis

**DOI:** 10.1093/ofid/ofae631.075

**Published:** 2025-01-29

**Authors:** Kori Keith, Gjermayne Wilson, Chrysovalantis Stafylis, Michael Reyes-Diaz, Kelika Konda, Jeffrey Klausner

**Affiliations:** University of Southern California Keck School of Medicine, Los Angeles, CA; AIDS Healthcare Foundation, Los Angeles, California; University of Southern California, Los Angeles, California; Universidad Peruana Cayetano Heredia, Lima, Lima, Peru; Universidad Peruana Cayetano Heredia, Lima, Lima, Peru; Keck School of Medicine of USC, Los Angeles, California

## Abstract

**Background:**

Syphilis has reemerged as a global health concern. The standard treatment for all stages of syphilis is injectable benzathine penicillin G. Cefixime is an FDA-approved, oral antibiotic widely available, low-cost, and safe in pregnancy. A previous pilot study showed cefixime was likely efficacious in treating early syphilis. This study aims to evaluate the efficacy of cefixime in comparison to benzathine penicillin G.

**Methods:**

We are conducting a randomized, multisite, open-label, non-inferiority clinical trial to compare the effectiveness of cefixime (400mg, orally, twice daily for 10 days) to benzathine penicillin G (2.4 million units, intramuscularly) in 400 participants from 12 clinical sites in the United States and Peru, including participants living with and without human immunodeficiency virus (HIV) infection. In the event of a penicillin shortage, participants randomized to penicillin receive doxycycline hyclate (100mg, orally, twice daily for 14 days).

Participants undergo follow-up visits post-treatment at 3-, 6-, and 9-months, involving clinical evaluation and rapid plasma reagin (RPR) testing. The primary outcome is a ≥4-fold decrease in the RPR compared to the initial RPR titer at enrollment, assessed at either 3 or 6 months after treatment.

**Results:**

As of May 1, 2024, 134 participants are enrolled, 67.2% living with HIV. The current cefixime adherence rate is 92.3% (60/65) at the 10 day mark. Evaluable data is available from 104 participants with 3- or 6-month follow-up visits, including 52 cefixime, 45 penicillin, and 7 doxycycline participants. By 3- or 6-month post-treatment, 88.5% (95% CI, 76.5% - 95.6%; 46/52) of cefixime participants, 88.9% (95% CI, 75.6% - 96.3%; 40/45) of penicillin participants, and 100.0% (95% CI, 59.0% - 100%; 7/7) of doxycycline participants achieved a ≥4-fold RPR titer decrease.

**Conclusion:**

Comparable effectiveness was observed among the treatment arms. Enrollment and data collection continue, with study completion expected by June 2026.

**Disclosures:**

**Jeffrey Klausner, MD MPH**, Direct Diagnostics: Advisor/Consultant

